# Age Related Changes in Muscle Mass and Force Generation in the Triple Transgenic (3xTgAD) Mouse Model of Alzheimer’s Disease

**DOI:** 10.3389/fnagi.2022.876816

**Published:** 2022-04-25

**Authors:** Hongyang Xu, Shylesh Bhaskaran, Katarzyna M. Piekarz, Rojina Ranjit, Jan Bian, Parker Kneis, Aubrey Ellis, Suyesha Bhandari, Heather C. Rice, Holly Van Remmen

**Affiliations:** ^1^Aging & Metabolism Research Program, Oklahoma Medical Research Foundation, Oklahoma City, OK, United States; ^2^OU Neuroscience, Graduate College and Department of Neurosurgery, University of Oklahoma Health Sciences Center, Oklahoma City, OK, United States; ^3^Oklahoma Center for Geroscience and Healthy Brain Aging, Department of Biochemistry and Molecular Biology, University of Oklahoma Health Sciences Center, Oklahoma City, OK, United States; ^4^Oklahoma City VA Medical Center, Oklahoma City, OK, United States

**Keywords:** Alzheimer’s disease, triple transgenic mice, amyloid-β (Aβ), sarcopenia, neuromuscular junction (NMJ)

## Abstract

Emerging evidence suggests that patients with Alzheimer’s disease (AD) may show accelerated sarcopenia phenotypes. To investigate whether pathological changes associated with neuronal death and cognitive dysfunction also occur in peripheral motor neurons and muscle as a function of age, we used the triple transgenic mouse model of AD (3xTgAD mice) that carries transgenes for mutant forms of APP, Tau, and presenilin proteins that are associated with AD pathology. We measured changes in motor neurons and skeletal muscle function and metabolism in young (2 to 4 month) female control and 3xTgAD mice and in older (18–20 month) control and 3xTgAD female mice. In older 3xTgAD mice, we observed a number of sarcopenia-related phenotypes, including significantly fragmented and denervated neuromuscular junctions (NMJs) associated with a 17% reduction in sciatic nerve induced vs. direct muscle stimulation induced contractile force production, and a 30% decrease in gastrocnemius muscle mass. On the contrary, none of these outcomes were found in young 3xTgAD mice. We also measured an accumulation of amyloid-β (Aβ) in both skeletal muscle and neuronal tissue in old 3xTgAD mice that may potentially contribute to muscle atrophy and NMJ disruption in the older 3xTgAD mice. Furthermore, the TGF-β mediated atrophy signaling pathway is activated in old 3xTgAD mice and is a potential contributing factor in the muscle atrophy that occurs in this group. Perhaps surprisingly, mitochondrial oxygen consumption and reactive oxygen species (ROS) production are not elevated in skeletal muscle from old 3xTgAD mice. Together, these results provide new insights into the effect of AD pathological mechanisms on peripheral changes in skeletal muscle.

## Introduction

Alzheimer’s disease (AD), the most common neurodegenerative disorder found in the elderly population, is typically characterized by the accumulation of extracellular amyloid plaques and intracellular neurofibrillary tangles in the brain (Querfurth and LaFerla, 2010). Amyloid plaques are primarily composed of the amyloid-β (Aβ) peptide, while hyperphosphorylated Tau is the primary constituent of intracellular neurofibrillary tangles (Jack et al., 2010). While the accumulation of Aβ in the brain is most commonly associated with AD, Aβ deposition and the expression of its precursor protein (amyloid precursor protein; APP) have also been detected in non-neural tissues, such as skeletal muscle, in both human and some animal models (Kuo et al., 2000; Monteiro-Cardoso et al., 2015). Moreover, progressive loss of skeletal muscle function, including a decline in muscle mass and strength, has been observed in human AD patients (Fukuchi et al., 1998; Burns et al., 2010; Ogawa et al., 2018). Abnormal loss of whole-body weight and cachexia are also characterized as clinical features of AD, although the underlying mechanisms are not fully understood (Ogawa et al., 2018). In fact, patients with AD have been reported to have a greater risk for sarcopenia than non-cognitively impaired age-matched controls (Sugimoto et al., 2016). In support of this, a study using MRI and DEXA found an increased loss in lean body mass associated with hippocampus atrophy, decreased cognitive ability, and a decreased brain volume in early-onset patients with AD compared to normal controls (Burns et al., 2010). In another study, decreased motor function and decreased grip strength, known risk factors for AD, were associated with mild cognitive impairment (Boyle et al., 2009). Together these studies strongly suggest that sarcopenia could be an intrinsic pathophysiologic effect of AD. Previously, our group has reported that skeletal muscle atrophy and weakness in aging are due to the denervation of muscle fibers (Larkin et al., 2011). However, to what extent AD pathology leads to degeneration of the neuromuscular junction (NMJ), denervation of skeletal muscles, and muscle atrophy and weakness remains unclear.

In this study, we utilized an AD mouse model that was created by inserting three mutant human genes into the mouse genome, APP, Tau, and PS1 known as the triple transgenic mouse model of AD (3xTgAD mice) (Oddo et al., 2003a). The expression of these mutated genes in the 3xTgAD mouse model recapitulates specific characteristics occurring in patients with AD, including age-dependent cognitive reduction, accumulation of amyloid plaques, and neurofibrillary tangles, as well as age-dependent inflammation (Janelsins et al., 2005; Belfiore et al., 2019). A few biochemical and metabolic deficiencies have previously been reported in skeletal muscle in 3, 6, and 12 months old 3xTgAD male mice, including impaired mitochondrial function and decreased enzymatic activities of antioxidant enzymes and reduced activity of acetylcholinesterase (Oddo et al., 2003a; Monteiro-Cardoso et al., 2015). Additionally, a recent study using the 3xTgAD mouse model reported gait impairments and loss of mobility, along with markers of muscle pathology, such as lower endurance, and smaller grip strength (Castillo-Mariqueo et al., 2021). However, the pattern of muscle atrophy and weakness and the functional changes related to NMJs and nerve-induced muscle force generation in 3xTgAD mice remain unknown.

Thus, the goal of this study was to investigate the effects of elevated expression of AD-related pathological proteins on the stability and morphology of the neuromuscular junction, muscle mass and strength, and muscle mitochondrial function in 3xTgAD mice in young (2.5 month) and older (18–20 months) female wildtype control and 3xTgAD mice. The incidence of AD has been reported to be higher in females (Vina and Lloret, 2010) and studies have indicated that female patients with AD are more susceptible to sarcopenia and physical inactivity (Ohta et al., 2019; Lee et al., 2020). Therefore, in this study, we measured changes in sarcopenia-related phenotypes in young and old female 3xTgAD mice. We observed an increase in muscle and NMJ impairments associated with sarcopenia in the older 3xTgAD mice compared to age-matched control mice. Mitochondrial function was measured in permeabilized muscle fibers in young and old control and 3xTgAD mice to determine whether the impairments seen in skeletal muscles are attributed to changes in mitochondrial metabolism. To gain insight into changes in muscle mass, we measured potential markers of atrophy activated by AD phenotypes in muscle. Together, our findings demonstrate that AD pathology can extend to motor neurons and alter skeletal muscle mass and function in older mice.

## Materials and Methods

### Triple Transgenic (3xTgAD) Mice

The triple transgenic (3xTgAD) mice used in this study have been previously described (Oddo et al., 2003a,b) and are maintained on a B6/129 background. This mouse model has been genetically engineered to contain homozygous mutations in three human genes associated with AD (PS1, APP, and Tau). The 3xTg-AD mice express the mutant human Tau protein, the human APP with the Swedish mutation and mutant presenilin. The mice were generated using two mutant transgenes (encoding APP_*swe*_ and Tau_*P*301*L*_ driven by the Thy1.2 promoter) microinjected into single-cell embryos harvested from PS1_*M*146*V*_KI mice (Oddo et al., 2003b). The 3xTg-AD mice progressively develop Aβ and Tau pathology, with a temporal- and regional-specific profile that closely mimics their development in the human AD brain, including progressive impairments in learning starting as early as 3 to 5 months, spatial memory deficits at 6 months, and recognition memory deficits at 9 to 11 months of age. Despite equivalent expression of the human APP and human Tau transgenes, Aβ deposition develops prior to the tangle pathology. Intracellular Aβ immunoreactivity is first detected around 4–6 months of age; extracellular Aβ deposits start to manifest around 12 months of age and as the mice age, plaques can be easily detected throughout the brain (Oddo et al., 2003a,b, 2006, 2007). Accumulation of intracellular Aβ is the first step of the events followed by Tau protein hyperphosphorylation, extracellular deposition of Aβ protein, and appearance of paired intracellular neurofilaments from hyperphosphorylated Tau (Oddo et al., 2003a; Mastrangelo and Bowers, 2008). As the mice age, Tau phosphorylation becomes more severe and can be detected by phospho-specific markers, such as AT8 and AT180 (Oddo et al., 2003b,^2007^). All mice were caged in a pathogen-free environment with free access to standard chow and water and maintained on a 12 h light/dark cycle. Measurements were done using young (2 to 4 months) and old (17–20 months) mice unless otherwise stated. The Institutional Animal Care and Use Committee at Oklahoma Medical Research Foundation (Oklahoma City, OK, United States) approved all procedures.

### Assessment of Muscle Contractile Properties, Functional Denervation, and Neuromuscular Junction Function *in situ*

Isometric contractile force generation was measured *in situ* in gastrocnemius (GTN) muscle based on the methods described previously (Larkin et al., 2011). Briefly, mice were anesthetized with isoflurane delivered by oxygen, and the whole GTN muscle was isolated and cleaned from surrounding muscles and connective tissues. After dissection, the isolated muscle was tied with a silk suture on the distal tendon, and the tendon was severed and mounted onto the force transducer (model 305B, Aurora Scientific). The mouse was placed on a temperature-controlled platform at 37°C and provided with continuous anesthesia. The electrode was placed on the surface of the GTN muscle directly, and the muscle optimal length was adjusted with single 0.2 ms stimulation pulses until a maximum twitch was reached. At the muscle optimal length, a series of 300 ms stimulus pulses were applied to achieve the maximum isometric tetanic force. After muscle stimulation, the electrode was moved from the muscle to the sciatic nerve, and the nerve filament was hooked firmly by the electrode. The same pulses of the tetanic stimulus were applied to the nerve to achieve the nerve-induced maximum isometric tetanic force. All of the above tetanic twitch protocols were repeated several times to confirm the reproducibility and reliability of the data. Comparing muscle and nerve stimulated contractile force generation allows us to measure the extent of denervation or loss of contractile force generation due to loss of intact NMJ innervation.

After all force measurements were completed, muscles were carefully removed and weighed, and the maximum tetanic force was normalized to the muscle cross-sectional area (CSA) calculated by the length and weight of the GTN muscle (dividing the muscle mass, mg, by the optimal length, mm, and the density of mammalian skeletal muscle, 1.06 g/cm^3^) to give the specific force (N/cm^2^). The NMJ function was presented by normalizing the nerve-induced force to the muscle-induced force as a percentage.

### Measurement of Mitochondrial Respiration and Reactive Oxygen Species Production

Mitochondrial function was measured in permeabilized muscle fibers. The permeabilization protocol has been previously described by our laboratory (Ahn et al., 2019). In general, a small piece of the red gastrocnemius muscle was excised from the body and finely dissected to separate the muscle fibers along their striations in cold *buffer X* containing (in mM): 7.23 K_2_EGTA, 2.77 CaK_2_EGTA, 20 imidazole, 0.5 DTT, 20 taurine, 5.7 ATP, 14.3 PCr, 6.56 MgCl_2_ -6H_2_O, and 50 K-MES (pH 7.1). A total of 30 μg/ml saponin was added to the fibers to induce permeabilization for 30 min, followed by 5 min washes for 3 times in washing buffer containing (in mM): 105 K-MES, 30 KCl, 10 K_2_HPO_4_, 5 MgCl_2_ -6H_2_O, 0.5 mg/ml BSA, 0.1 EGTA (pH 7.1). After washing, the permeabilized fibers were placed into the Oxygraphy-2K (O2k, OROBOROS INSTRUMENTS, Austria) following the protocols described before (Krumschnabel et al., 2015). The oxygen consumption rate (OCR) was determined using the oxygen probe, while the ROS production rate was measured by the O2K-Fluo LED2-Module Fluorescence-Sensor Green with Amplex UltraRed Reagent (Invitrogen, A36006). Substrates used for different complexes added sequentially as follows, leak state: 10 mM glutamate and 2 mM malate with no ADP; complex I: addition of 2.5 mM ADP; complex I + II: addition of 10 mM succinate; complex II: addition of 0.5 μM rotenone; complex IV: addition of 2 mM ascorbate and 0.5 mM TMPD added after 5 μM antimycin A. An H_2_O_2_ standard curve was measured each time before the actual experiments to calibrate the level of H_2_O_2_. All data generated from O2K was normalized to the muscle wet weights and analyzed using the official O2K software, Datalab Version 7.0.

### Contractile Proteins Composition Assay

Skeletal muscle contractile proteins (myosin heavy chain (MHC) and actin) composition was determined by SDS-PAGE gel. In this study, gastrocnemius muscle samples were homogenized and denatured as described previously (Xu et al., 2017, 2018). Then the denatured whole muscle sample was separated with a 10% SDS-PAGE gel. Then the gel underwent electrophoresis at 200 V at room temperature for 1 h, and immediately after running, Coomassie brilliant blue G250 was used to stain the gel and visualize the MHC bands. The images were collected by G:BOX Chemi (Syngene, United States), and the densitometry analysis was done for the ratio between MHC and actin, and also the proportion of MHC and action together to the total proteins. The densitometry analysis was conducted using ImageJ software (ImageJ, Fiji).

### Confocal Microscopy for Neuromuscular Junction Morphology

After the muscle was excised from the body, small muscle pieces were taken under the cold PBS in a petri dish along with the fiber directions with careful removal of fat and connective tissues. Muscle samples were transferred into a 24-well plate with 10% STUmol in deionized water (Poly Scientific R&D, #2832) for 1 h to fix the tissue by gently shaking. After the fixation, tissues were washed 3 times for 5 min in PBS at room temperature, then permeabilized tissue under the 2% Triton in PBS for 30 min on a shaker. After permeabilization, the tissues are placed into the blocking buffer containing 4% BSA, 1% Triton, and 5% serum, matching the host of secondary antibody diluted in PBS, blocking overnight in the cold room at 4°C. After blocking, we added primary antibodies, 1:50 SV2 (DSHB) for nerve terminals, and 1:50 2H3 (DSHB) for neurofilaments. The tissues were incubated overnight at 4°C, washed 6 times for 30 min in PBS at room temperature, and subsequently incubated with the secondary antibodies, 1:1,000 BTX-Alexa 488 (Invitrogen, #B13422), and 1:250 goat anti-mouse Cy3. After the incubation for secondary antibodies overnight at 4°C, all tissues were then washed 6 times for 30 min with PBS. Afterward, tissues were transferred onto slides and mounted with a mounting medium, and sealed with nail polish. Images of NMJ were taken by the Nikon confocal microscope under the magnification of 20×, and Z-stacks were taken to show the 3D structure of intact NMJ. The total thickness of optical sections is around 20 to 60 μm, and the stack interval was set at every 1 to 2 μm, so there are around 20 to 30 images per stack.

The NMJ area was analyzed as the area occupied by each individual labeled acetylcholine receptor (ACHR), and only the NMJs facing forward were analyzed. The fragmentation level was quantified by counting the pieces of each ACHR, and if there are five or more pieces per junction, the NMJ is considered a fragmented NMJ. The denervation score was quantified as follows: score 0: there is no denervation, the ACHRs fully overlap with nerve terminals; score 1: partial denervation, the ACHR partially overlaps with nerve terminals; score 2: complete denervation, there is little or no overlap of ACHR and nerve terminals. The statistical analysis for all images taken in this section was done by ImageJ software (ImageJ).

### Quantitative Real-Time PCR (qRT-PCR)

qRT-PCR was performed as described before (Bhaskaran et al., 2020). In brief, total RNA was isolated from 25 mg gastrocnemius muscle using Trizol, and cDNA was synthesized using the iScript cDNA kit (Bio-Rad, United States). qRT-PCR was performed using Power SYBR green PCR mix. The following PCR primers were used: TGFβ (forward- CCCTATATTTGGAGCCTGGA), TGFβ (reverse- CTTGCGACCCACGTAGTAGA), 18S rRNA (forward- GTGGAGCGATTTGTCTGGTT), 18S rRNA (reverse- CGCTGAGCCAGTCAGTGTAG). Calculations were performed by the comparative (2^ΔΔ*Ct*^) method using 18S rRNA.

### Western Blotting and Antibody Information

Gastrocnemius muscles were homogenized in RIPA buffer containing 50 mM Tris (pH 7.4), 150 mM NaCl, and protease inhibitors. Then the total protein was quantified with the Bio-Rad protein assay kit (Sigma-Aldrich, Poole, United Kingdom), and the same amount of protein was loaded and separated with SDS-PAGE gels at certain percentages, i.e., 10 or 12%. The gel was then run at 200 V for 1 h and wet-transferred onto 0.45 μm nitrocellulose membranes (Bio-Rad) with the conditions of 100 V, 30 min at 4°C, same as described before (Murphy et al., 2012). After the transfer of the gel, total proteins in each lane were quantified using ponceau staining (Sigma, #P3504), then the membrane was washed with ddH_2_O to remove the ponceau staining and blocked with 1% BSA solution in TBST for at least 1 h at room temperature. Soon after blocking, primary antibodies were added onto the membrane and incubated overnight at 4°C. After the incubation of the primary antibody, the membrane was washed with blocking buffer and then exposed to the secondary antibody for 30–60 min. After the secondary antibody, the membrane was washed with TBST for the last time to clean the background. Protein bands were visualized and quantified using the Gene tool system (SynGene-Frederick, MD, United States). The relative content of each protein measured using western blot analysis was normalized to sample total protein content measured using ponceau stain and densitometry of total ponceau in that sample lane. Primary antibody information, rabbit anti-Amyloid Precursor Protein (APP) (Abcam, ab32136), rabbit anti Tau (Abcam, ab76128), chicken anti-TGF-β1/1.2 (R&D systems, AF-101-NA), rabbit anti-Smad 2/3 (Cell Signaling, #3102), rabbit anti-phospho-Smad2/3 (Cell Signaling, #8828), Smad2/3 pure protein (cell extract) (Cell Signaling, control: #26725, phosphorylated: 47986), mouse anti MuRF1 (Santa Cruz, sc-398608 HRP).

### Aβ Extraction and Multi-Plex Electrochemiluminescence Immunoassay

Samples were obtained from the brain, gastrocnemius muscle, and spinal cord and mechanically homogenized in 7.5 volumes of ice-cold TBS with phosphatase and protease inhibitors. Following centrifugation at 55,000 rpm for 1 h at 4°C, the supernatant (TBS extract) was collected. The pellet was then resuspended and mechanically homogenized in 7.5 volumes of TBS with 1% Triton X-100 containing protease and phosphatase inhibitors. Following centrifugation at 55,000 rpm for 1 h @ 4°C, the supernatant (TBS-T extract) was collected. The pellet was then resuspended in 2 volumes of 88% formic acid and sonicated at 4°C for 1 min and gently agitated on a shaking platform at 4°C overnight. The formic acid extract is sonicated at 4°C for 1 min, clarified by spinning for 10 min at 1,800*g* at 4°C, and neutralized by adding non-buffered Tris 1 M, pH11.

Aβ42 and Aβ40 concentrations in TBS, TBS-T, and formic acid extracts were measured by multi-plex electrochemiluminescence immunoassay using Aβ peptide panel 1 (6E10) kit (Cat # K15200E-2) according to manufacturer’s instructions (Mesoscale Discovery). The kit was supplied with peptides (Aβ42 and Aβ40), diluent 35 and 100, SULFO-TAG Anti-Aβ 6E10 antibody, read buffer T, and MSD plates. The samples were thawed on ice and diluted 2 times in diluent 35. Eight standards were prepared by dissolving 10 μl of each peptide in 370 μl of diluent 35 and four-fold serial dilutions. Diluent 35 (150 μl) was added to each well of the MSD plate and incubated in a platform shaker at 700 rpm and at room temperature for 1 h. All subsequent incubation steps were carried out similarly. The antibody solution was prepared with 60 μl of SULFO-TAG Anti-Aβ 6E10 antibody in 2,940 μl of Diluent 100. After incubation, wells were washed thrice with PBS-0.05% tween solution followed by the addition of 25 μl of antibody solution. A 25 μl of prepared samples and standards were added to the wells and incubated for 2 h. Read buffer was prepared by diluting read buffer T 2 times in deionized water. Wells were washed thrice with PBS-0.05% tween solution followed by adding 150 μl of the read buffer. The plates were read immediately after the addition of reading buffer with MSD Quickplex SQ 120.

### Statistical Analysis

All results are presented as mean values ± standard error of the mean (SEM), and comparisons among different groups were performed with one-way ANOVA and Tukey’s multiple comparison test. The statistical analysis was completed using GraphPad Prism 8, and the statistical significance was set at *p* values less than 0.05.

## Results

### Tissue and Age-Specific Protein Levels of Amyloid Precursor Protein, Tau, and Aβ in Female 3xTgAD Mice

To characterize the expression of APP and Tau transgenes in neural and muscle tissue of young and old control and 3xTgAD female mice we measured APP and Tau protein in homogenates from the brain, spinal cord, sciatic nerve, and gastrocnemius muscle by western blotting (Supplementary Figure 1). While APP expression was elevated in brain tissue of both young and old 3xTgAD compared to controls, APP expression in the spinal cord, sciatic nerve, and gastrocnemius muscle was increased only in old 3xTgAD mice compared to controls (Supplementary Figures 1B,D,F,H). Tau expression was elevated in the brain, spinal cord, and sciatic nerve tissue from both young and old 3xTgAD female mice compared to wild-type mice (Supplementary Figures 1A,C,E). Tau expression was not different between groups in gastrocnemius muscle tissue. We also measured APP and Tau expression in male 3xTgAD mice and age-matched control mice, and although we had a small sample size (*n* = 3) we did not observe a significant increase in the amount of these two proteins in male 3xTgAD mice, in agreement with other reports (Mendell et al., 2020).

**FIGURE 1:**
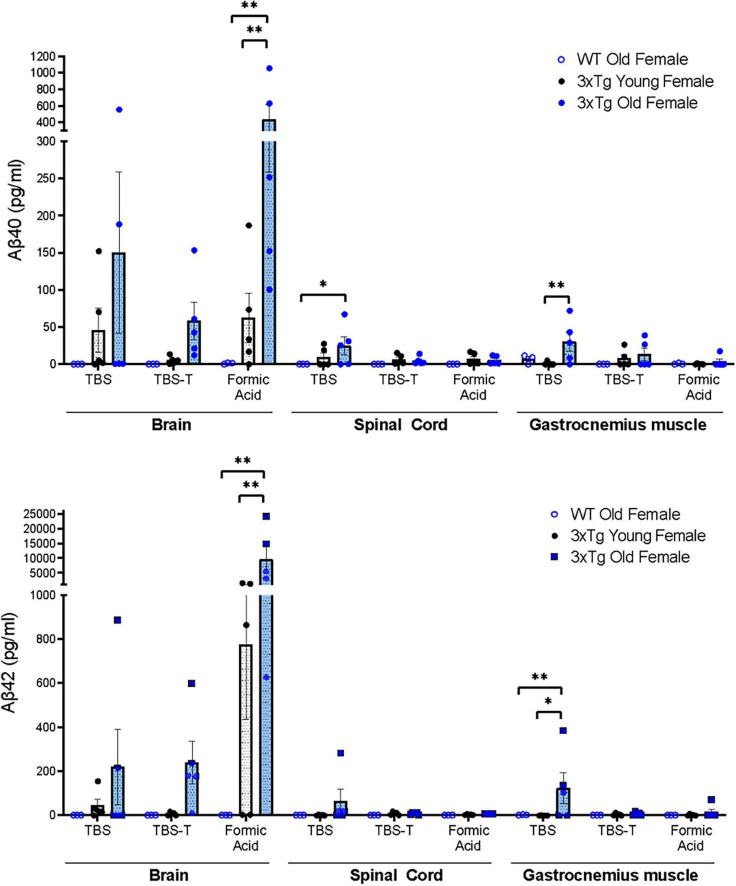
Quantification of Aβ40 and Aβ42 measured by multiplex immunoassays (meso-scale discovery) from TBS soluble, TBS-T soluble, and Formic Acid soluble fractions of homogenates of brain, spinal cord, and gastrocnemius muscle tissue homogenates (Two-way ANOVA) *n* = 3–5, indicating the number of animals. Data are presented as Mean ± SEM.

To determine whether soluble and insoluble forms of Aβ accumulate in neural and muscle tissue of 3xTgAD mice, we performed multiplex immunoassays (meso-scale discovery) to measure Aβ40 and Aβ42 from TBS, TBS-T, and formic acid-soluble fractions from the brain, spinal cord, and gastrocnemius muscle of old and young 3xTgAD mice (and old wildtype as control) (Figure 1). As expected, no human Aβ40 and Aβ42 were detected in any of the fractions of wild-type mice. In the 3xTgAD mouse brain, Aβ40 and Aβ42 both increased with age, particularly insoluble Aβ found in formic acid-soluble fractions. Strikingly, Aβ40 and Aβ42 were detectable in the TBS soluble fraction of both spinal cord and gastrocnemius muscle of old female 3xTgAD mice, and in gastrocnemius muscle their amounts were significantly elevated compared to young female 3xTgAD mice (Figure 1). The large variation observed in the Aβ amount might be due to normal biologic variability.

### Muscle Atrophy Is Evident in Gastrocnemius From 17 to 20 Months Old 3xTgAD Mice but Not in Age-Matched Wild-Type Mice

**FIGURE 2:**
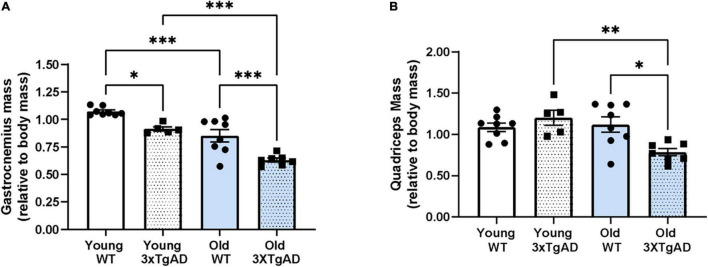
Mass of gastrocnemius and quadriceps muscles from both legs in control and 3xTgD mice is normalized to the total body weight. **(A,B)** are the gastrocnemius and quadriceps muscle mass relative to body mass. * indicates significant difference between the labeled groups (*p* < 0.05, unpaired *t*-test). *n* = 5–9, indicating the number of animals. Data are presented as Mean ± SEM.

We measured the mass of the gastrocnemius and quadriceps muscle (normalized to body weight) in the hindlimb of young and old wildtype and 3xTgAD mice. Body mass is increased with age in both genotypes but was not different between wildtype and 3xTgAD mice (22.4 ± 0.6 vs. 19.4 ± 0.4 g for young female wildtype vs. 3xTgAD mice and 31.8 ± 3.6 vs. 40.0 ± 1.3 g in the old control and 3xTgAD mice). The normalized muscle weight was significantly decreased in gastrocnemius muscles from both young (15%) and old (26%) female 3xTgAD mice compared to age-matched wildtype control mice (Figure 2A). Quadriceps muscle mass was not reduced in the older wildtype mice but was significantly lower in older 3xTgAD mice (Figure 2B).

### Neuromuscular Junction (NMJ) Function Is Impaired

**FIGURE 3:**
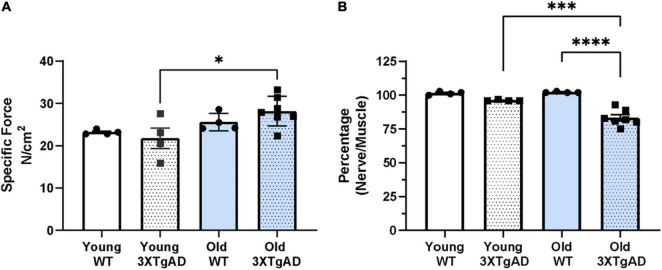
Specific muscle maximum force **(A)** obtained from stimulating gastrocnemius muscle from wildtype and 3xTg mice. Normalized proportion of the maximum nerve derived force through stimulating sciatic nerve to the maximum muscle-derived force **(B)**. * indicates significant difference between the labeled groups (*p* < 0.05, one-way ANOVA). *n* = 4–7, indicating the number of animals. Data are presented as Mean ± SEM.

To determine whether the reduction in mass was also accompanied by a loss in function, we measured the muscle maximum specific force and the function of the neuromuscular junction (NMJ) in young and old female mice using *in situ* electrical stimulation of the sciatic nerve. Maximum specific force production was not different between wildtype and 3xTgAD female mice (Figure 3A). The integrity of NMJ function was determined by normalizing the nerve-induced force when stimulated to the muscle-induced force as a proportion (an intact normal NMJ should be able to trigger a nerve-induced force close to 100% of the muscle-induced force). The NMJ function in old 3xTgAD mice was significantly reduced by 17% of the nerve-induced force when compared with wild-type mice (Figure 3B). NMJ function in young 3xTgAD mice was preserved with no significant reduction in nerve-induced force production (Figure 3B). Together these data indicate that muscle strength was not affected by AD phenotypes, but NMJ function was impaired in older female 3xTgAD mice compared to wild-type mice.

### Morphological Measurement of the Neuromuscular Junction Indicates Significant Fragmentation and Denervation Occurred in Old Female 3xTgAD Mice

**FIGURE 4:**
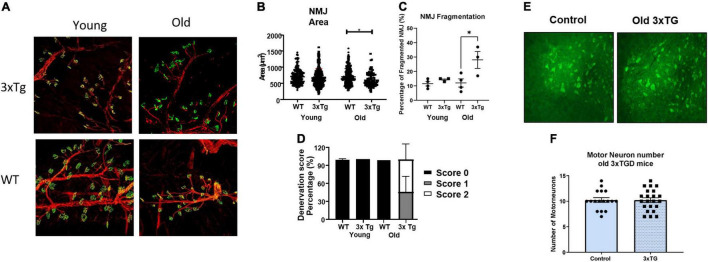
Representative confocal images for NMJ **(A)** and motor neurons **(E)**. **(B–D)** Quantified data for NMJ area, fragmentation, denervation for female old and female young, and **(F)** are quantified data for motor neuron numbers. * indicates significant difference between the labeled groups (*p* < 0.05, one-way ANOVA). *n* = 3–4, indicating the number of animals. Data are presented as Mean ± SEM.

To directly assess the morphology of the NMJ, neurofilaments (red) and acetylcholine receptors (ACHRs, green) were stained and examined by confocal microscopy, and NMJ area, degree of fragmentation, and denervation score were measured. NMJ morphology was altered, with significant denervation and fragmentation, in the old female 3xTgAD mice compared to age-matched controls (Figure 4A). The percentage of fragmented NMJs is 30% in old female 3xTgAD mice, which is dramatically higher than its age-matched wildtype (10%) (Figure 4C), with the majority of NMJ being partially or completely denervated (Figure 4D). In contrast, the fragmentation and denervation were similar in wild-type mice and the young 3xTgAD mice (Figures 4C,D). In addition, the measurement of NMJ area did not show a difference between old wildtype and 3xTgAD (Figure 4B), however, in the young mice, this area was reduced in 3xTgAD mice (602 μm^2^) compared to wildtype mice (700 μm^2^) (Figure 4B). These data show that the NMJ dysfunction in the old female 3xTgAD muscles is related to NMJ morphological alterations. To determine whether the NMJ phenotype is related to changes in motor neuron decline in the spinal cord, we measured the number of alpha-motor neurons in the lumbar region of the spinal cord sections using the NeuN antibody (Figure 4E). There was no change in the number of alpha motor neurons (Figure 4F).

### Oxygen Consumption Rate (OCR) and the Generation of Reactive Oxygen Species Are Not Altered in Old Female 3xTgAD Mice

**FIGURE 5:**
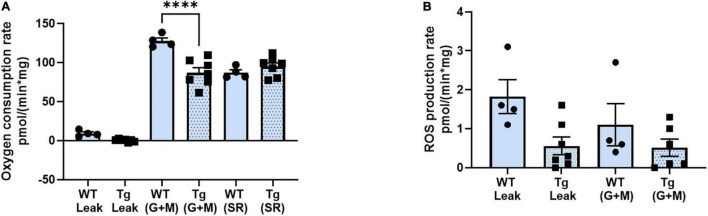
Respiratory rate (oxidation-phosphorylation rate) **(A)**, and the reactive oxygen species (ROS) production rate of fibers at different stages **(B)**. * indicates significant difference between the labeled groups (*p* < 0.05, one-way ANOVA). *n* = 4–7, indicating the number of animals. Data are presented as Mean ± SEM.

It is well known that loss of innervation and NMJ dysfunction induce elevated mitochondrial dysfunction and generation of mitochondrial peroxides in muscle (Muller et al., 2007; Arthur et al., 2008; Pharaoh et al., 2020). To determine whether the changes in NMJ function and morphology observed in old female 3xTgAD mice altered muscle mitochondrial function, we measured oxygen consumption rate (OCR) and ROS generation as peroxide emission in permeabilized muscle fibers from gastrocnemius muscle from old female wildtype and 3xTgAD mice. Using respiratory substrates to stimulate electron flow through different electron transport chain complexes, we found a decrease in OCR in transgenic compared to wildtype mice using glutamate/malate, complex I linked substrates but not in response to complex II using succinate rotenone (Figure 5A). There was no difference in State 1 (leak state, mitochondria respiring without the addition of external substrate) or complex I (mitochondria respiring with the addition of glutamate and malate) ROS production rate (Figure 5B). Overall, these data suggest that mitochondrial function may be mildly impaired in the old female 3xTgAD mice but not at a level that induces elevated mitochondrial ROS generation.

### The Relative Amount of Contractile Proteins and the Ratio Between Actin and Myosin Are Not Altered in Old or Young Female 3xTgAD Mice

**FIGURE 6:**
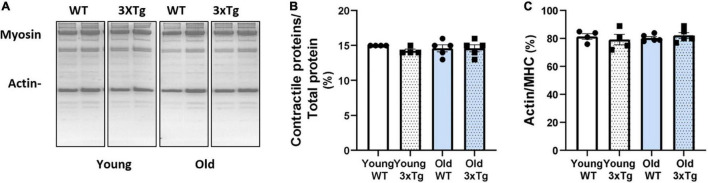
Representative SDS-PAGE gel images showing the myosin and actin with the total protein in different groups as labeled **(A)**. Pooled data are showing the proportion of contractile proteins over total protein **(B)**, and the ratio between actin and myosin **(C)** in female old young groups, respectively. *n* = 4–5, indicating the number of animals. Data are presented as Mean ± SEM.

To determine whether there are differences in the contractile protein composition of the muscle, we measured the abundance of contractile proteins, myosin and actin, relative to total muscle protein in whole muscle homogenates from gastrocnemius muscle by using SDS-PAGE gel (Figure 6A). When normalized to total protein, the relative proportion of contractile proteins was not different in young or old 3xTgAD female mice compared to their age-matched wildtype mice (Figure 6B). Moreover, there is no difference between the ratio of actin to myosin, in young or old 3xTgAD female mice compared to age-matched wild-type mice (Figure 6C). These data show that the basic contractile components of skeletal muscle were not affected even in the old female 3xTgAD mice.

### TGF-β Atrophy-Related Signaling Pathway Is Activated in Old 3xTgAD Gastrocnemius Muscle

**FIGURE 7:**
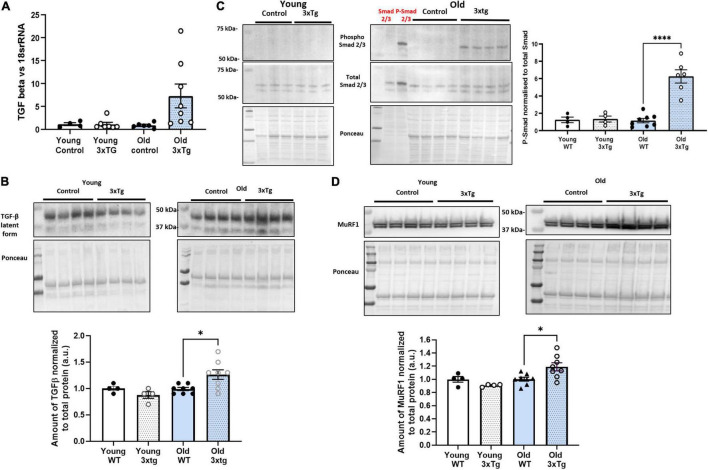
TGF-β atrophy-related signaling pathway is activated in old 3xTgAD gastrocnemius muscle. Muscle samples from both wildtype and 3xTg mice in young and old female mice were detected. Transcription level was detected by qPCR, and protein level was detected by western blotting. **(A)** qPCR data showing the transcription level of TGF-β in females old and young. **(B)** Representative western blot images with pooled data for TGF-β in females old and young. **(C)** Are the images with pooled data showing the amount of phospho Smad 2/3, total Smad 2/3, and the data of phospho Smad2/3 normalized to total Smad 2/3 across different groups in female (old and young) mice. **(D)** Are the images with pooled data showing the amount of MuRF1 in different groups in female (old and young) mice. * indicates significant difference between the labeled group (*p* < 0.05, one-way ANOVA and unpaired *t*-test). *n* = 4–8, indicating the number of animals. Data are presented as Mean ± SEM.

It has previously been reported that the amount of TGF- β is elevated in neural tissues with AD (Kinney et al., 2018). In skeletal muscle, TGF-β downstream proteins such as Smad and MuRF1 are closely associated with muscle atrophy (Sartori et al., 2009; Bollinger et al., 2014; Kinney et al., 2018). In order to determine if the TGF-β associated muscle atrophy pathway is altered in 3xTgAD mice, we measured the level of TGF-β transcription in gastrocnemius muscle from young and old female wildtype and 3xTgAD mice. TGF-β mRNA is elevated in old female 3xTgAD mice compared to age-matched control and young mice (Figure 7A). Consistent with mRNA level, a similar increase in TGF-β protein levels (∼30%) was observed in muscle from old female 3xTgAD vs. wildtype mice, with no change detected in young mice (Figure 7B).

Smad 2/3 is a protein involved in TGF-β signaling that is activated by phosphorylation and is associated with muscle atrophy (Sartori et al., 2009). Phosphorylation of Smad 2/3 in gastrocnemius muscle was undetectable by western blotting in old female wildtype and both wildtype and 3xTgAD in young mice but was apparent in 3xTgAD old mice (Figure 7C). MuRF1 is a downstream effector protein of Smad 2/3 (Bollinger et al., 2014). MuRF1 levels were significantly increased in old female 3xTgAD mice (∼25%) but were unchanged in young female 3xTgAD mice compared to controls (Figure 7D). Together, these data confirmed that the TGF- β associated muscle atrophy pathway is activated in old female 3xTgAD muscle, and is a potential effector of the decreased muscle mass seen in the gastrocnemius muscle in old female 3xTgAD mice. We also measured the TGF-β signaling pathway in male 3xTgAD and control mice, and both mRNA and protein levels of TGF-β are not different between 3xTgAD and wildtype mice, and Smad 2/3 phosphorylation is not elevated. The level of MurF1 protein is also the same in male 3xTgAD and wild-type mice (Supplementary Figure 4).

## Discussion

The progression of Alzheimer’s disease is associated with a general loss of vitality in addition to the loss of memory and cognition. Recently, the progression of AD has been correlated with reduced skeletal muscle mass, strength, and function (Ogawa et al., 2018). Understanding the impact of AD on peripheral motor neurons and skeletal muscle mass and function is the first step to interventions that might alleviate adverse peripheral effects in older AD patients and provide some benefit for quality of life. While the pathologic effects of Aβ and Tau in the brain are well documented, we know very little about the effects of these proteins on the peripheral nervous system and skeletal muscle. It is reasonable to predict that peripheral neurons and muscle would show similar sensitivity to the effects of Aβ and Tau and might be adversely affected. In this study, we hypothesized that the pathological proteins and pathways that cause neuronal damage and cognitive decline in Alzheimer’s disease can also potentially exacerbate sarcopenia, an age-related disease initiated by neuromuscular junction decline (denervation) and its downstream impacts on skeletal muscle mass and function. Our results show a clear effect of elevated expression of AD-associated proteins on the NMJ and skeletal muscle with age in the 3xTgAD mouse model of Alzheimer’s disease. For the first time, our findings show the systematic impact of mutant APP and Tau on skeletal muscle mass and function in older mice.

While the effect of Aβ and Tau in the AD brain has been extensively studied, the role of these proteins in the spinal cord, peripheral nervous system, and muscle and in tissue from older mice has not been well defined. In this study, we used the 3xTgAD mouse model, which develops both amyloid and Tau pathology, and studied the effect of transgene expression in young and old mice. While a number of studies have utilized the 3xTgAD mouse model, very few have measured phenotypes present in 3xTgAD mice after 12 months of age. The impact of age-related changes could potentially be very important, as the occurrence of sporadic AD is closely associated with age, and AD phenotypes are more severe at an older age (Griffith et al., 2019; Arsenault et al., 2020). As widely reported in the 3xTgAD mouse model, we found a high expression of APP and Tau protein in brain tissue. However, we also observed elevated APP, soluble Aβ40, and Tau in the spinal cord of aged female 3xTgAD mice compared to controls. Our findings in the spinal cord are in agreement with previous studies reporting increased levels of Aβ in the spinal cord in AD mouse models. For example, Seo et al. (2010) found increased levels of Aβ in the spinal cord in the Tg2576 AD mouse model associated with significant spinal deficits and reduced spinal cord mitochondrial function beginning at 10 months of age. Likewise, Yuan et al. (2017) found increased levels of Aβ in the spinal cord of the TgCRND8 double mutation AD mouse model which was associated with motor function deficits. Increased Aβ has also been found in the spinal cord of ALS patients (Calingasan et al., 2005). In addition to the spinal cord, we also observed a significant accumulation of APP, Aβ40, Aβ42, and Tau in gastrocnemius muscle of aged female 3xTgAD mice compared to controls. In particular, the Aβ level in gastrocnemius muscle was measured by ELISA assay in a human-specific way, hence, its accumulation in the muscles is more likely generated from the transgene in the central neural system (CNS) rather than from the endogenous APP, however, we cannot be absolutely certain of the cell-type of origin due to the methodological limitation. Our findings agree with a previous study by Arai et al. (1991) that reported deposition of Aβ42 in muscle from AD patients, and Kuo et al. (2000) reported Aβ42 in muscle from autopsy patients. Levels of elevated APP have also been reported in skeletal muscle in APP/PS1 mice (Schuh et al., 2014). Together these studies support the potential for pathological effects of APP, Aβ and Tau in tissues other than brain that may contribute to the increased loss of muscle mass and function seen in AD patients.

It is reasonable to hypothesize that accumulation of Aβ and Tau in motor neurons or muscle from old 3xTgAD mice may contribute to deleterious muscle phenotypes. Consistent with this idea, Aβ accumulation in muscle tissue is associated with muscle atrophy in inclusion body myopathy (Fukuchi et al., 1998) and in a recent study in a mouse model with Aβ accumulation (Tg2576 mice), Torcinaro et al. (2021) reported reduced cholinergic innervation of skeletal muscle that potentially contributes to sarcopenia. Tau overexpressing mice (Tg30 mice) also show motor dysfunction in mice at 8 months of age, including axonopathy, muscle atrophy, and decreased NMJ innervation (Audouard et al., 2015). Interestingly, we observed a significant decrease in muscle mass, but not strength, in old 3xTgAD mice compared to age-matched control mice. Muscle atrophy can be initiated by a number of causes including loss of innervation, inactivity, or diseases, and often but not always occurs concurrently with muscle weakness. Our data showing that the contractile protein abundance and the ratio of actin to myosin are not compromised in the old 3xTgAD mice compared to age matched wildtype mice, suggests that there was no major loss of sarcomeric proteins and is consistent with no increase in muscle weakness in the older 3xTgAD mice.

While the maximum specific force was not compromised in the gastrocnemius muscle from the old 3xTgAD mice compared to age-matched control mice, we did find evidence of functional denervation or a reduction in nerve stimulated vs. direct muscle stimulated force generation consistent with a decline in neuromuscular transmission and integrity of the NMJ. Using confocal microscopy, we found that old 3xTgAD mice showed increased denervation and fragmentation in their NMJs. In contrast, young 3xTgAD mice show no change in NMJ morphology. APP is expressed at the neuromuscular junction (Akaaboune et al., 2000) and APP proteins have been shown to be required for the proper development and function of the NMJ (Torroja et al., 1999; Wang et al., 2005). Lrp4, an NMJ-associated protein, and APP have been shown to bind to each other and APP can also bind agrin to promote AchR clustering (Choi et al., 2013). Thus, it is possible that altered levels of APP or Aβ in the peripheral motor neurons could alter NMJ structure and function and contribute to loss of muscle mass and contractile function. Consistent with changes in NMJ morphology and enhanced denervation, we observed an increased accumulation of APP and Tau proteins in the spinal cord and sciatic nerve from old but not young 3xTgAD mice compared to age-matched control mice.

Denervation not only induces loss of muscle mass and function but is also associated with the elevated mitochondrial generation of peroxides, which can elicit further damage to mitochondrial function (Muller et al., 2007) and contribute to muscle degeneration. We found only mild changes in mitochondrial respiration and no change in peroxide generation in permeabilized muscle fibers from the old 3xTgAD vs. age-matched wild-type mice. These findings are consistent with a relatively mild extent of denervation (only 15%) in the 3xTgAD mice.

**FIGURE 8:**
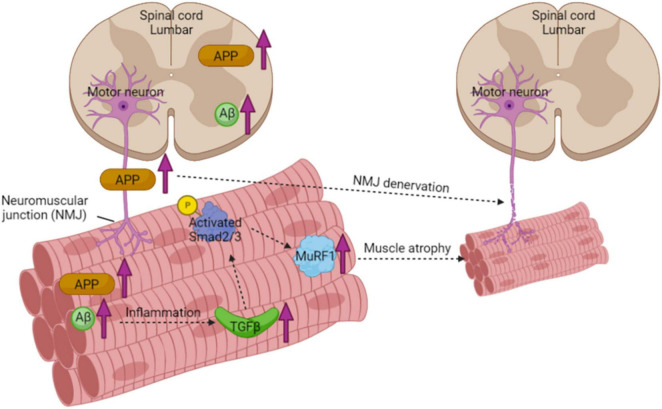
Schematic diagram of muscle atrophy pathway in muscle from 3xTgAD mice. Increased expression of APP in the spinal cord and sciatic nerve results in fragmented and denervated NMJ. The higher amount of Aβ found in skeletal muscle causes inflammation with upregulating TGFβ, excessive amount of TGFβ then activates Smad2/3 through phosphorylation. Activated Smad2/3 then upregulates its downstream effector protein MuRF1, which is a muscle-specific ubiquitin ligase and sequentially inhibits the protein turnover in skeletal muscles and hence causes the decrease of muscle mass.

To explore the underlying mechanisms of elevated muscle atrophy in the 3xTGAD mice, we measured the expression of transforming growth factor-β (TGF-β). TGF-β mediates pleiotropic effects on different cell systems including adipose and connective tissues (Lee, 2004; Wagner et al., 2008) and is believed to play an important role in muscle impairment (Mann et al., 2011). In skeletal muscle, it modulates satellite cell activation and differentiation and functions as a negative regulator of muscle growth. In particular, TGF-β and a number of related proteins are closely associated with muscle atrophy pathways, e.g., Smad and MuRF1 (Lee, 2004; Sartori et al., 2009; Bollinger et al., 2014; Kinney et al., 2018). It has been well documented that in AD, TGF-β is significantly elevated in cerebrospinal fluid, serum, and brain microvascular endothelial cells (Kinney et al., 2018). Based on that, our present study, for the first time, detected both the mRNA and protein levels of TGF-β in skeletal muscle and found an increase in old female 3xTgAD muscle only. The upregulation of TGF-β leads to phosphorylation and activation of downstream transcription factors, Smad 2 and Smad 3 (Sartori et al., 2009), which we confirmed in muscle of old 3xTgAD female mice. The activated Smad 2/3 (phosphorylation) is then able to induce an independent atrophy program through upregulating muscle RING-finger protein 1 (MuRF1), a muscle-specific ubiquitin ligase, which will sequentially inhibit the protein turn over in skeletal muscles and hence cause the decrease of muscle mass (Sartori et al., 2009). Therefore, we measured the amount of MuRF1 in muscles from all different groups, and found an increase in muscle from old 3xTgAD compared to age matched control mice, consistent with the changes we measured in Smad 2/3 and TGF-β. Overall, our finding support that the muscle atrophy observed in old 3xTgAD mice is strongly associated with TGF-β mediated Smad-MurF atrophic signaling pathway (Figure 8).

Importantly, our findings that 3xTgAD female mice have more severe muscle atrophy, NMJ fragmentation, and denervation than male mice is consistent with other studies that found more severe neuropathological phenotypes in females compared to the male 3xTgAD mice (Yang et al., 2018). This finding is also in accordance with the effects of AD phenotypes in humans, where females are at a greater risk of developing dementia than males with AD (Podcasy and Epperson, 2016). However, in contrast to our findings, a previous report by Monteiro-Cardoso et al. (2015) used only male 3xTgAD mice and reported reduced ACHE enzyme activity and reduced mitochondrial function in muscle. As shown in the Supplementary Data, we did not observe a loss of muscle or nerve stimulated force generation in old male 3xTgAD mice (16–18 months old) and the NMJ morphology was normal. The sex difference in this line could be due to the widespread use of the 3xTgAD models and the generation of many different sublines, with each of them presenting the onset and progression of AD-associated pathology differentially, which has then led to reported differences between sexes and ages (Belfiore et al., 2019). Therefore, these findings show that a clear record of the mouse strain, age, and sex are critical for the use of 3xTgAD mice.

## Conclusion

In summary, the results in this study clearly demonstrate the effects of AD pathology on skeletal muscle from the perspectives of functions and metabolisms, including muscle mass and strength, mitochondrial and nerve functions, in different sexes and ages. We observed muscle atrophy and NMJ disruption in muscles from only old female 3xTgAD mice, along with the TGF-β mediated atrophic signaling pathway is activated in their skeletal muscles as well. We detected Aβ in skeletal muscle, and its abnormal accumulation in old 3xTgAD muscles potentially contributes to muscle atrophy and nerve dysfunction. Together, our findings systematically document alterations in skeletal muscles between males and females at different ages in the 3xTgAD mouse model. Together, our findings provide insight into the underlying mechanisms of how muscle function and metabolisms are affected by AD pathologies, which will further contribute to the prevention or treatment of sarcopenia in patients with AD.

## Data Availability Statement

The datasets presented in this study can be found in online repositories. The names of the repository/repositories and accession number(s) can be found in the article/Supplementary Material.

## Ethics Statement

The animal study was reviewed and approved by the Institutional Animal Care and Use Committee at Oklahoma Medical Research Foundation.

## Author Contributions

HX, ShB, and HVR contributed to the conception and design of the research. HX, ShB, KP, RR, JB, PK, AE, SuB, and HR performed the experiments and analyzed the data. HX, ShB, HR, and HVR interpreted the results of the experiments, prepared the figures, drafted the manuscript, edited, and revised the manuscript. All authors approved the final version of the manuscript, contributed to the article, and approved the submitted version.

## Conflict of Interest

The authors declare that the research was conducted in the absence of any commercial or financial relationships that could be construed as a potential conflict of interest.

## Publisher’s Note

All claims expressed in this article are solely those of the authors and do not necessarily represent those of their affiliated organizations, or those of the publisher, the editors and the reviewers. Any product that may be evaluated in this article, or claim that may be made by its manufacturer, is not guaranteed or endorsed by the publisher.
