# Supplementary material for: Age Related Changes in Muscle Mass and Force Generation in the Triple Transgenic (3xTgAD) Mouse Model of Alzheimer’s Disease
Source: Front Aging Neurosci. 2022 Apr 25;14:876816. doi: 10.3389/fnagi.2022.876816 (PMC9083113; doi:10.3389/fnagi.2022.876816)
Supplement: Supplementary file 1 [file Data_Sheet_1.docx]

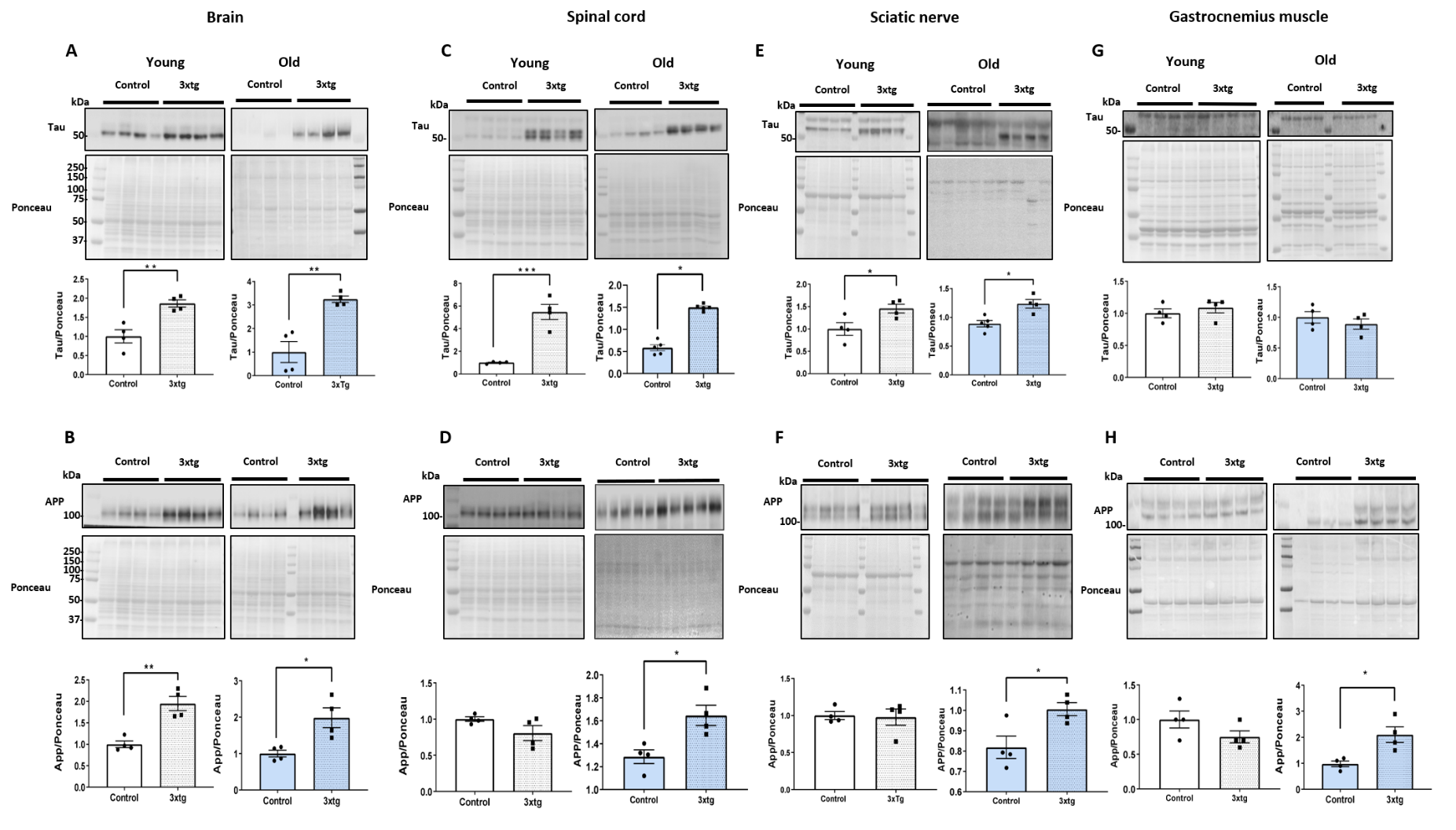


**SFigure 1.** Representative western blot images and pooled data showing the level of App4 and Tau protein in different tissues, including brain, spinal cord, sciatic nerve, and gastrocnemius muscle, in young (3-4 month) and old (17-26 month) female wildtype and 3xTgAD mice. (A) to (D) are the images with pooled data for App4 protein, and (E) to (G) are the images and pooled data for Tau protein. * indicates significant difference between the labelled groups (*P*<0.05, One-Way ANOVA). *n*=4-5, indicating the number of animals. Data are presented as Mean ±SEM.


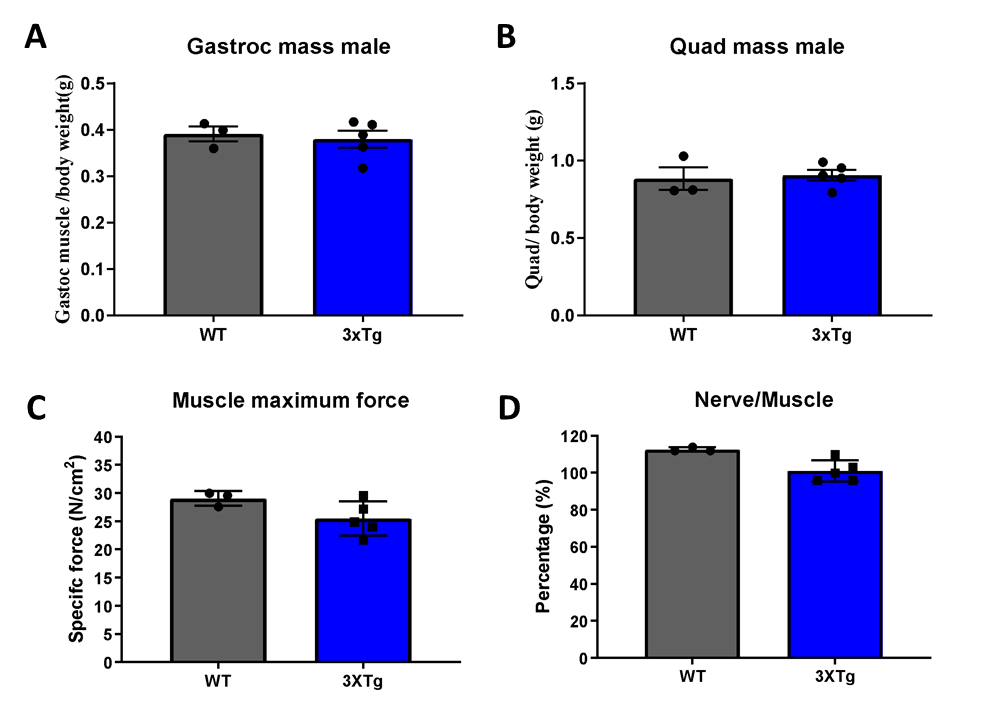


**SFigure 2**. Mass of gastrocnemius (A) and quadriceps (B) muscles from both legs in male WT and 3xTgD mice is normalized to the total body weight. *n*=3-5, indicating the number of animals. Specific muscle maximum force obtained from stimulating gastrocnemius muscle from male WT and 3xTg mice (C). (D) shows the normalized proportion of the maximum nerve derived force through stimulating sciatic nerve to the maximum muscle derived force. *n*=3-5, indicating the number of animals. Data are presented as Mean ±SEM.


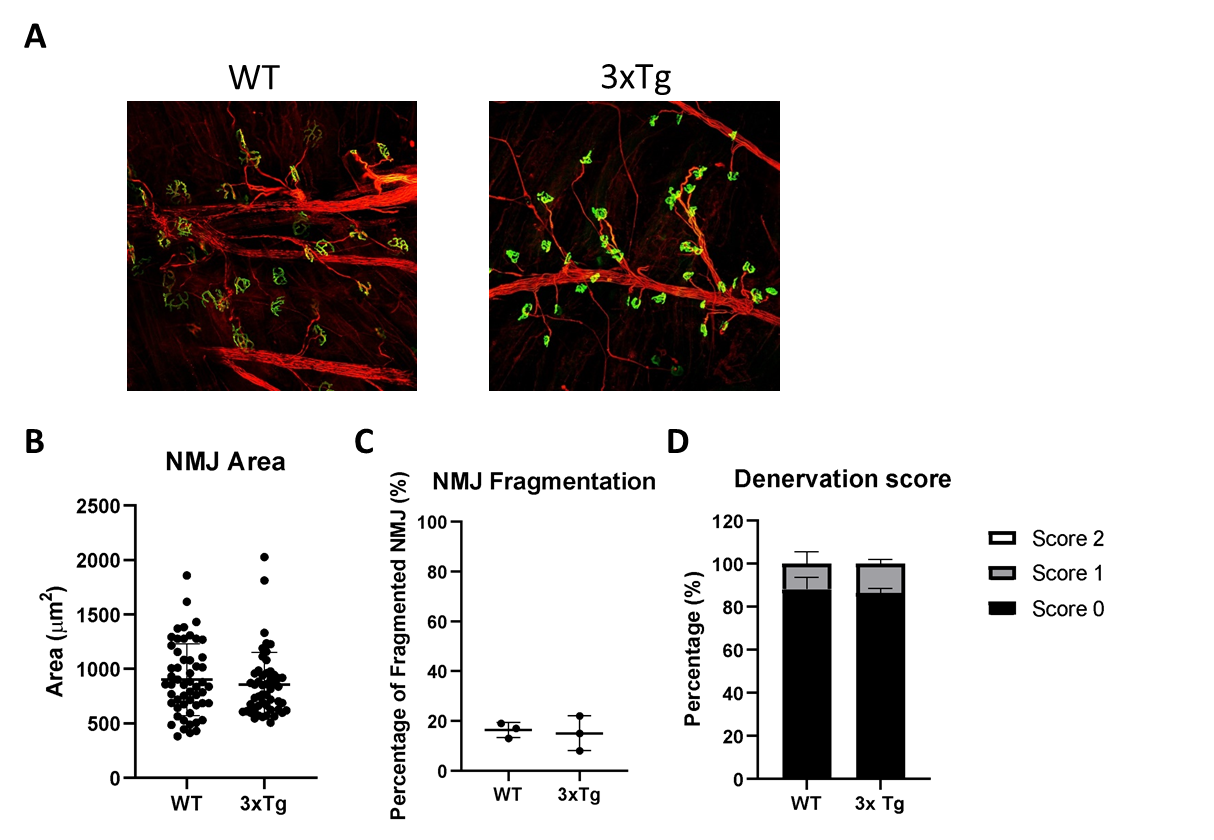


**Sfigure 3.** Representative confocal images for NMJ in male WT and 3xTg groups as labelled (A). (B), (C), and (D) are pooled data for NMJ area, fragmentation, and denervation score. *n*=3, indicating the number of animals. Data are presented as Mean ±SEM.


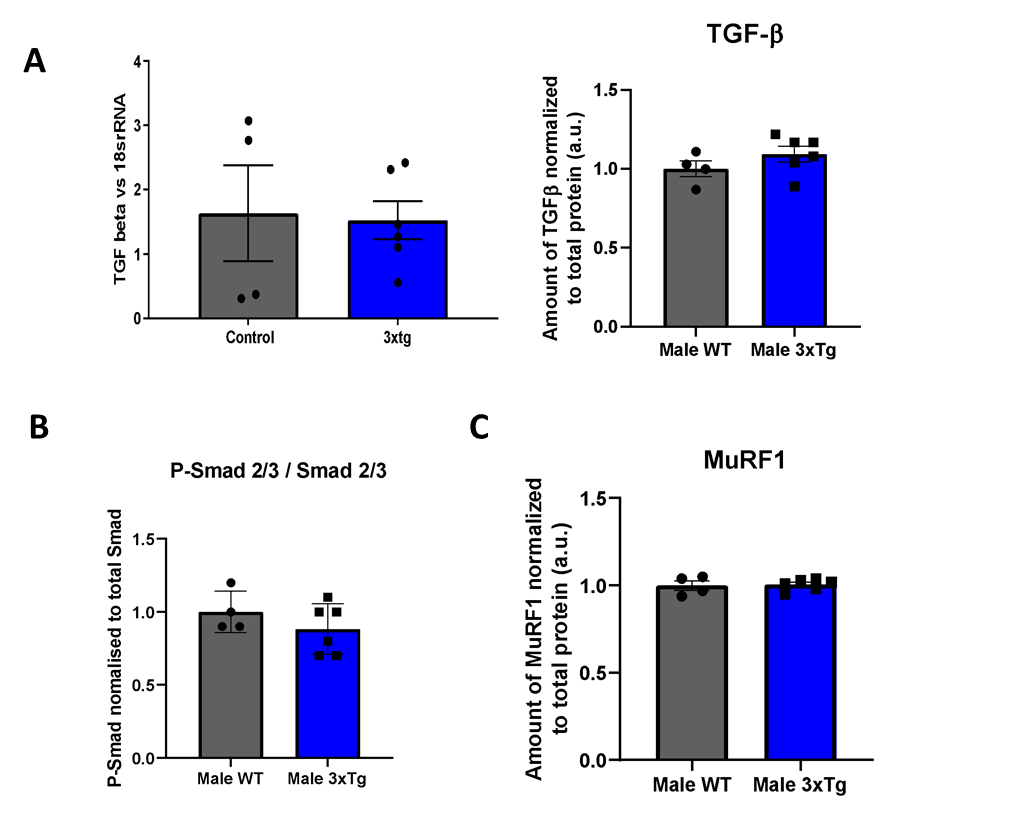


**SFigure 4.** Transcription and protein level of TGF-β in gastrocnemius muscle in male WT and 3xTg mice. Muscle samples from both WT and 3xTg mice in male groups were detected. Transcription level was detected by qPCR, and protein level was detected by western blotting. (A), qPCR data showing the transcription level of TGF-β in male WT and 3xTg muscles respectively; (B), representative western blot images with pooled data for TGF-β in males WT and 3xTg. *n*=4-6, indicating the number of animals. Data are presented as Mean ±SEM. Representative western blot images and pooled data showing the activation of Smad 2/3 proteins and its downstream protein MuRF1 in gastrocnemius muscle in male mice. Muscle tissues were from both WT and 3xTg mice in male WT and 3xTg groups. (A) are the images with pooled data showing the amount of phospho Smad 2/3, total Smad 2/3, and the data of phospho Smad2/3 normalised to total Smad 2/3 across different groups in mice. (B) are the images with pooled data showing the amount of MuRF1 in different groups in male mice. *n*=4-6, indicating the number of animals. Data are presented as Mean ±SEM.


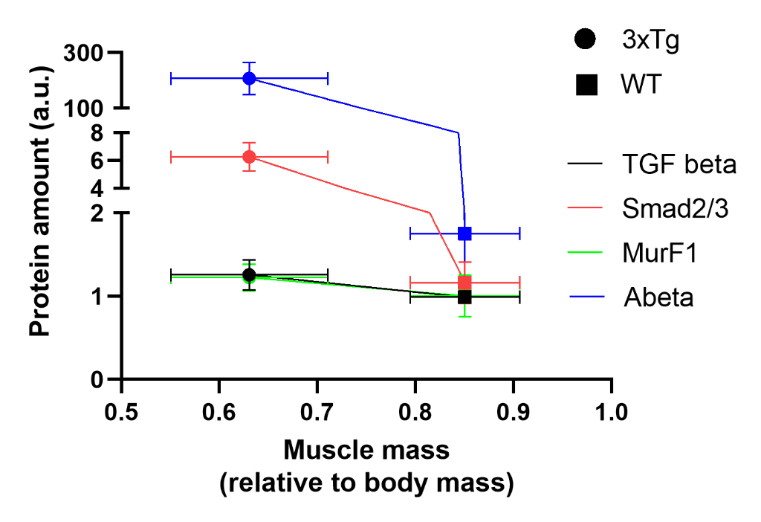


**SFigure 5.** Correlations between muscle mass and protein amounts related to Aβ and TGF-β pathways, in gastrocnemius muscle from old female WT and 3xTgAD mice. Round and square symbols stand for 3xTgAD and WT groups respectively, and symbols and connective lines in different colours represent different proteins as indicated in the legend.
